# AffyRNADegradation: control and correction of RNA quality effects in GeneChip expression data

**DOI:** 10.1093/bioinformatics/bts629

**Published:** 2012-10-24

**Authors:** Mario Fasold, Hans Binder

**Affiliations:** ^1^Interdisciplinary Center for Bioinformatics, Universität Leipzig, D-4107 Leipzig, Haertelstr. 16-18, Germany and ^2^Leipzig Research Center for Civilization Diseases, Universität Leipzig, Leipzig, Germany

## Abstract

**Motivation:** Gene expression experiments aim to accurately quantify thousands of transcripts in parallel. Factors posterior to RNA extraction can, however, impair their accurate representation. RNA degradation and differences in the efficiency of amplification affect raw intensity measurements using Affymetrix expression arrays. The positional intensity decay of specifically hybridized probes along the transcript they intend to interrogate is used to estimate the RNA quality in a sample and to correct probe intensities for the degradation bias. This functionality, for which no previous software solution is available, is implemented in the R/Bioconductor package AffyRNADegradation presented here.

**Availability:** The package is available via Bioconductor at the URL http://bioconductor.org/packages/release/bioc/html/AffyRNA Degradation.html

**Contact:** Fasold@izbi.uni-Leipzig.de

**Supplementary information:** Supplementary data are available at *Bioinformatics* online.

## 1 INTRODUCTION

A basic assumption in gene expression experiments is that the obtained data represent a snapshot of transcript abundances within the original sample. However, several effects can distort the amount of RNA during sample extraction and preparation, and thereby impair the reliability of those measurements. RNases introduced by improper purification or incautious sample handling can degrade the rather unstable RNAs during storage (Fleige and Pfaffl, 2006). Also, the amplification of RNA mandatory to most RNA analytics differs in its efficiency and can therefore lead to variation in transcript yield and lengths (Ma *et al.*, 2006).

Gene expression experiments are frequently conducted using high-density microarrays. Because of the importance of RNA quality for the reliability of the results, it is advised to check the integrity of the RNA before hybridization to the array. RNA integrity (RIN) values (Schroeder *et al.*, 2006) that are determined on the basis of an electropherogram trace have become the standard measure of RNA quality. Samples with RIN values >7 should be discarded.

Researchers increasingly conduct large-scale meta-analysis on the plethora of publicly available microarray data. For these data, RNA quality measures are mostly not available. However, it is strongly advised to identify and to remove low RNA-quality experiments, as they can lead to erroneous results. Methods to estimate RNA quality directly from microarray data are thus required. Existing options are the use of 3′/5′ intensity ratios of control probe sets included on the microarray, as well as 3′/5′-summary degradation measures as provided by software tools such as the affy package (Gautier *et al.*, 2004). Both methods have been shown to have drawbacks under circumstances that are relevant in large-scale studies (Fasold and Binder, 2012). Particularly, 3′/5′ control probes might be affected by saturation, whereas affyslope estimates are affected by background hybridization. Both methods are prone to systematically overestimating RNA quality.

Beyond strict quality control and the removal of bad-quality samples, the continuous levels of RNA quality transform into a gray area of biased expression results with questionable reliability. It has been previously found that, although moderate levels of RNA degradation are tolerated by differential expression analysis, especially long targets provide erroneous results.

In this work, we present an R package that assesses RNA quality of Affymetrix expression data. It provides a RNA quality measure that overcomes the drawbacks of existing methods by strictly referring to specific hybridization. Furthermore, it enables correction of the 3′ probe intensity bias for improved downstream analysis.

For illustration, we here use data from an experiment done by Archer *et al.* (2006) where the same cell extract has been used for multiple microarray hybridizations, however, either prepared with RNeasy to remove RNA degrading enzymes, or not.

## 2 FUNCTIONALITY

On Affymetrix 3′, expression arrays up to 16 probes of length 25 nt interrogate each transcript. Most of these probes cover a specific region located within 600 nt distance to the 3′ end of the transcripts. RNA samples are usually prepared using an *in vitro* transcription labeling and amplification assay with primers starting at the 3′ poly-A tail of the source mRNA. Both degradation of mRNA as well as effectiveness of the amplification assay are thus captured by multiple probe measurements for each transcript.

### 2.1 Analyzing RNA degradation and amplification

**Fig. 1.**
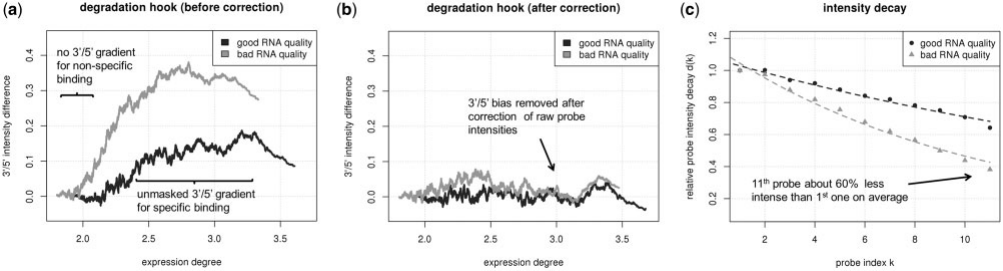
Degradation hook plots referring to strongly and weakly degraded RNA taken from Archer *et al.* (2006) before [panel (**a**)] and after [panel (**b**)] correction using AffyRNADegradation. The height of the hook curve increases with increasing degradation level. Panel (**c**) shows the respective probe positional decays d(x) as plotted by the AffyRNADegradation package: the worse the RNA quality, the steeper is the respective decay

Limited RNA quality of a given sample leads to intensity differences between probes located at the 3′ end and those located closer toward the 5′ end of the mRNA. The so-called degradation hook-plot, shown in Figure 1a and b, displays this 3′/5′ intensity difference in dependence on the mean logged probe intensity approximating the expression degree of the respective gene. Cross-hybridization of partly matching targets of other genes causes nearly equal intensities for weakly expressed genes (Binder, 2006). With increasing expression competitive binding of specific targets progressively unmasks their actual 3′/5′ gradient, until probe saturation sets in. Desirable would be equal intensities for 3′ and 5′ probes for all expression levels. The maximum height of the hook-plot reflects the relevant 3′/5′-intensity gradient of the selected array enabling the unbiased comparison of differentially expressed genes under variable RNA quality.

The hook-plot is accessible using the PlotDegradationHook function in the package. A complementary representation is the Tongs Plot shown in the Supplementary Material and accessible using the PlotTongs function.

### 2.2 Estimation of the RNA quality of a sample

One should only use specifically hybridized probes for estimation of RNA quality because of the 3′/5′ gradient of the intensity as a function of the expression degree. For these probes, we compute the mean probe intensity separately for each probe index k = 1. . .11 starting from the 3′ end of the target transcript. Figure 1c shows the resulting probe positional intensity decay after normalization with respect to the mean intensity for the first probe k = 1. Alternatively, the intensity decay can be calculated as a function of the distance L of the probes given in units of nucleotides from the 3′-transcript end (not shown).

We determine the decay-length parameter d from the mean intensity decays of all specifically hybridized probes. It provides an accurate estimate for the RNA quality of a particular array hybridization improving other array-based metrices (Fasold and Binder, 2012). The d(x = k,L) plot is available via the PlotDx function, and the RNA quality estimate is available via the d function in the AffyRNADegradation package.

### 2.3 Correcting the RNA quality bias

Differences in RNA quality and the resulting probe positional intensity decay are technical artifacts that can affect expression measures and the results of differential expression analysis. Microarray experiments are often subject to such RNA quality variation (Upton *et al.*, 2009).

We here aim at removing the systematic differences in probe positional intensities between different conditions. Figure 1a shows two such conditions in the example data relating to degraded transcripts due to increased presence of RNases not removed by RNeasy treatment. AffyRNADegradation first estimates specific probes based on the degradation hook-plot described above. It then uses a correction function that reverses the probe positional intensity decay d(x) after applying the expression level dependency of the hybridization mode (details are given in the Supplementary Material). Optionally, the correction can be performed based on probe indices k as well as probe distances L. Differences between both options are discussed in the Supplementary Material and in (Fasold and Binder, 2012). Figure 1b shows the degradation hook after correction using probe indices k: The 3′/5′ bias is almost completely removed. Corrected probe intensities are available via the afbatch function.

### 2.4 Package usability

The AffyRNADegradation package extends the Bioconductor package affy and integrates well in a typical microarray analysis workflow. All calculations are performed directly on the AffyBatch object and carried out separately for each particular microarray hybridization in a single-chip approach. Our approach corrects the 3′/5′-bias on the level of raw probe intensities, which can afterward be processed with any method. The runtime is about 2 min and 3 min per sample for index and distance based corrections, respectively. Because each chip is processed independently, arbitrarily large data sets can be processed.

## Supplementary Material

Supplementary Data
