# Supplementary material for: AffyRNADegradation: control and correction of RNA quality effects in GeneChip expression data
Source: Bioinformatics. 2012 Oct 24;29(1):129–31. doi: 10.1093/bioinformatics/bts629 (PMC3530908; doi:10.1093/bioinformatics/bts629)
Supplement: Supplementary Data [file supp_29_1_129__index.html]

AffyRNADegradation: control and correction of RNA quality effects in GeneChip expression data — Supplementary Data 

# AffyRNADegradation: control and correction of RNA quality effects in GeneChip expression data

## Supplementary Data

files

**Files in this Data Supplement:**

- Supplementary Data - pdf file
