# Supplementary material for: AffyRNADegradation: control and correction of RNA quality effects in GeneChip expression data
Source: Bioinformatics. 2012 Oct 24;29(1):129–31. doi: 10.1093/bioinformatics/bts629 (PMC3530908; doi:10.1093/bioinformatics/bts629)
Supplement: Supplementary Data [file supp_bts629_AffyRNADegradation-supplement.pdf]

Supplementary text

AffyRNADegradation: Control and correction of RNA quality effects in GeneChip expression data

Mario Fasold[1,2]* and Hans Binder[1,2]

[1] Interdisciplinary Center for Bioinformatics; Universität Leipzig, D-4107 Leipzig, Haertelstr. 16-18
[2] Leipzig Research Center for Civilization Diseases; Universität Leipzig, Germany

* Corresponding author: E-mail: fasold@izbi.uni-leipzig.de, fax: ++49-341-9716679

Table of contents

1. Tongs plot and degradation hook.................................... 2
2. Probe positional intensity decays.................................... 2
3. Correcting the probe positional intensity bias................. 3
4. Positional information of the probes............................... 4
5. Choosing between absolute and relative probe positions. 4
6. References 4

1. Tongs plot and degradation hook

We present two graphical representations that allow assessing the degradation of RNA-transcripts in a chip-specific fashion. These so-called ‘degradation hook’ and ‘tongs plot’ estimate the 3’-enrichment of the probes and thus their degradation level in dependence on the expression degree. They depict the mean intensity difference between two selected subsets of perfect-match probes taken from the 3’- and 5’-ends of each probe set, respectively,

$$\Delta = \Delta\Sigma_{s3'/s5'} \equiv \left\langle \Sigma_p \right\rangle_{s3'} - \left\langle \Sigma_p \right\rangle_{s5'} \quad \text{(degradation hook)}$$

$$\Delta\Sigma_s \equiv \left\langle \Sigma_p \right\rangle_s - \left\langle \Sigma_p \right\rangle_{pset} \quad \text{(tongs plot)} \quad ,$$

$$\text{with} \quad \left\langle \Sigma_p \right\rangle_s \equiv \frac{1}{3}\sum_{k=i}^{i+2} \log I_k^{PM}$$

as a function of the average logged intensity $y = \Sigma \equiv \left\langle \Sigma_p \right\rangle_{pset}$ of all probes within the probe sets which estimates the expression degree to a rough approximation. The subscript s= s3’, s5’ assigns the respective subsets of three consecutive probes from either the 3’ or the 5’ end of the probe set. Three probes are chosen from each side of the probe sets to ensure robust averaging over their intensities *and* to ensure sufficiently large differences between the averaged intensities of both subsets, and thus a proper tradeoff between robustness and sensitivity. Note that the number of three probes refers to about 1/3 to 1/4 of the size of each probe set of typically 11 probes for most array types. On the other hand, our choice is not crucial: Selecting subsets of two or four probes from both ends of the probe sets only marginally alters the results (data not shown).
Figure S 1 shows the tongs plot and degradation hook for the same two samples that were used also in the main paper. In general, the degradation hook is more suited to compare the degradation between different samples. The tongs plot reveals additional information such as an asymmetrical behavior of the 3’ and 5’ subsets.

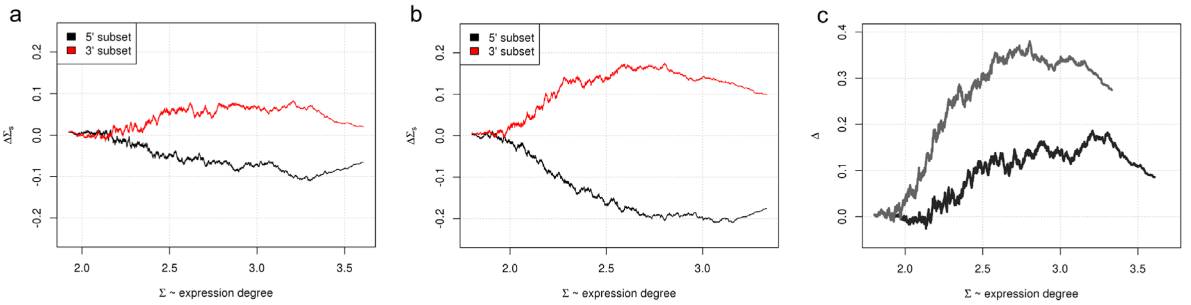

Figure S 1. Tongs plots (Panel a and b) and degradation hooks (panel c) of two array hybridizations using either weakly or strongly degraded RNA: With progressing degradation the ‘tongs opening’ (i.e. the maximum gap between the red 3’ and black 5’ branches) and the height of the hook increase. The two branches of the tongs plot and the two different hook curves converge at small abscissa values owing to the insensitivity of non-specific hybridization for degradation effects. The curves of both hybridizations are slightly shifted from each other in horizontal direction due to different scanner settings. The samples are taken from ref. (Archer et al., 2006) (samples VOV1_GOOD.CEL and VOV1_INHIBITED.CEL, respectively).

2. Probe positional intensity decays

Two main factors related to RNA quality potentially affect the intensities of the probes : (i) the distance of a probe relative to the 3’-end of the transcript, L (or, alternatively, the probe index in the probe set, k, which counts the probes in direction away from the 3’-end of the transcript) and (ii), the hybridization mode (Fasold and Binder, 2012). In the specific (S-) hybridization mode the probes bind amplified RNA (aRNA) fragments of complementary sequence originating from the mRNA

transcripts which they intend to detect. In the N-hybridization mode the probes bind to aRNA fragments of partly complementary sequence originating however from other mRNA transcripts not referring to the interrogated gene. We normalize the intensity of probes at position x= k, L with respect to the intensity of probes located at the 3'-end to obtain the degradation index

$$d^h(x) = I^h(x) / I^h(3') \quad \text{with} \quad x = k, L \quad \text{and} \quad h = N, S \ ,$$

where $I^h(x)$ is the average perfect-match probe intensity of all probes with hybridization mode h=N or S and the same index k=1...11 (for x=k) or the same position L on the current array. For x=L, we use all probes in windows of +/-25 bases about the absolute positions L=25, 75, …, 575 (typically more than 95% of all probes are located within the range L=1..600). $I^h(3')$ is the average intensity level of the probes near the 3' end, i.e. $I^h(3')= I^h(k=1)$ and $I^h(3')= I^h(1<L<50)$ for x=k and x=L, respectively.

The degradation index due to non-specific hybridization is virtually constant $d^N(x)\approx 1$. The degradation index due to specific hybridization shown in Figure 1 of the main paper is described using an exponential decay of the form

$$d(x) = d^S(x) \approx \left(1 - d_\infty^x\right) \cdot \exp\left(-\frac{x - x_0}{\lambda_x}\right) + d_\infty^x$$

3. Correcting the probe positional intensity bias

The raw probe intensities of each sample are corrected as follows:

1. The degradation hook $\Delta\Sigma_{3'/5'}$-vs-Σ is calculated using all perfect-match probe intensities for the current array as described in section 1.
2. Probes are considered as specifically hybridized if the sigma-value of the respective probe set meets the condition $\Sigma_{3'/5'}^{max} - 0.4 < \Delta\Sigma_{3'/5'} < \Sigma_{3'/5'}^{max} + 0.2$ where $\Sigma_{3'/5'}^{max} = \arg\max\left\{\Delta\Sigma_{3'/5'}(y)\right\}$.
3. The decay function $d^S(x)$ (x=k, L) is calculated as described in section 2 using the subensemble of all specifically hybridized probes.
4. The mean fraction of probe intensities due to specific hybridization is estimated for each probe set as, $f^S(y) = \Delta\Sigma_{3'/5'}(y) / \Delta\Sigma_{3'/5'}^{max}$.
5. The correction function is calculated as weighted sum of the decay functions due to specific and non-specific hybridization where the latter one is simply set to unity, $d^N(x)= 1$, i.e. $$C(x, y) = d^S(x) \cdot f^S(y) + d^N(x) \cdot \left(1 - f^S(y)\right) = d^S(x) \cdot f^S(y) + \left(1 - f^S(y)\right)$$
6. The biased probe intensities are then corrected using the inverse of the correction function, $I_p^{P,x-corr} = I_p^P / C(x, y)$.

Note that each probe intensity is rescaled according to value of the mean intensity decay at its position (x= k or L) and according to its hybridization mode as indicated by the abscissa-value of its probe set y. Consequently, probe intensities taken from the non-specific hybridization range remain uncorrected. With increasing degree of specific hybridization the probes are progressively scaled up with increasing distance from the 3'-end of the transcript. The maximum correction applies to probe sets in the S-hybridization range. MM probe intensities are scaled using the mean logged MM-intensity of the probe set as argument.

4. Positional information of the probes

The distances of the probes to the 3' end are obtained by aligning their sequences to the respective transcript sequence serving as target for the respective probe set as provided by Affymetrix. We have computed files containing probe positional information for a large number of GeneChip expression arrays. They are available via the website http://www.izbi.uni-leipzig.de/downloads_links/programs/rna_integrity.php.

These files are stored in R binary file format. The package documentation contains a section describing the contents of this file and explaining how the user can easily create and use custom probe location files, for example if he uses custom microarrays.

5. Choosing between absolute and relative probe positions

In the supplementary text of ref. (Fasold and Binder, 2012) we compare the two correction metrics based either on the absolute probe position ('L-correction') or on the relative probe position (index-based, 'k-correction') relative to the 3′ transcript end. The k-correction applies the same positional factor to all probe sets. In consequence, the probe set-specificity of the correction is solely determined by the degree of specific hybridization. Contrarily, the L-correction applies a specific factor to each probe-set depending on the particular location of its probes. Comparison of both correction methods shows that probe sets located on the average nearer to 3′-end of the transcript are corrected to a less degree using their absolute position than probe sets located more distant from the 3′-transcript end. Hence, the L-correction is more specific with respect to each particular probe set. On the other hand, the k-correction is more robust with respect to outliers.

We recommend use of absolute probe positions to cope with the effect of differently distributed probes. In practice the intensity changes due to index-based and position-based correction differ only slightly with, in general, small differences in the resulting expression values.

6. References

Archer,K.J. et al. (2006) Assessing quality of hybridized RNA in Affymetrix GeneChip experiments using mixed-effects models. *Biostatistics*, **7**, 198-212.

Fasold,M. and Binder,H. (2012) Estimating RNA-quality using GeneChip microarrays. *BMC genomics*, **13**, 186.
